# Anti-liver fibrosis activity of curcumin/chitosan-coated green silver nanoparticles

**DOI:** 10.1038/s41598-022-23276-9

**Published:** 2022-11-01

**Authors:** Alya Elzoheiry, Esraa Ayad, Nahed Omar, Kadry Elbakry, Ayman Hyder

**Affiliations:** 1 Zoology Department, Faculty of Science, Damietta University, New Damietta, Egypt; 2 Faculty of Science, Damietta University, New Damietta, 34517 Egypt

**Keywords:** Biotechnology, Physiology, Medical research, Nanobiotechnology

## Abstract

Liver fibrosis results from the hepatic accumulation of the extracellular matrix accompanied by a failure of the mechanisms responsible for matrix dissolution. Pathogenesis of liver fibrosis is associated with many proteins from different cell types. In the present study, in silico molecular docking analysis revealed that curcumin may inhibit the fibrosis-mediating proteins PDGF, PDGFRB, TIMP-1, and TLR-9 by direct binding. Nano-formulation can overcome curcumin problems, increasing the efficacy of curcumin as a drug by maximizing its solubility and bioavailability, enhancing its membrane permeability, and improving its pharmacokinetics, pharmacodynamics and biodistribution. Therefore, green silver nanoparticles (AgNPs) were synthesized in the presence of sunlight by means of the metabolite of *Streptomyces malachiticus,* and coated with curcumin-chitosan mixture to serve as a drug delivery tool for curcumin to target CCl_4_-induced liver fibrosis mouse model. Fibrosis induction significantly increased hepatic gene expression of *COL1A1, α-SMA, PDGFRB, and TIMP1*, elevated hepatic enzymes, increased histopathological findings, and increased collagen deposition as determined by Mason’s trichrome staining. Treatment with naked AgNPs tended to increase these inflammatory effects, while their coating with chitosan, similar to treatment with curcumin only, did not prevent the fibrogenic effect of CCl_4_. The induction of liver fibrosis was reversed by concurrent treatment with curcumin/chitosan-coated AgNPs. In this nano form, curcumin was found to be efficient as anti-liver fibrosis drug, maintaining the hepatic architecture and function during fibrosis development. This efficacy can be attributed to its inhibitory role through a direct binding to fibrosis-mediating proteins such as PDGFRB, TIMP-1, TLR-9 and TGF-β.

## Introduction

In chronic liver injury, fibrogenesis arises as a non-adaptive inflammatory response characterized by a cascade of events resulting in recruitment of inflammatory cells to the injured liver, activation of collagen-producing cells, and excessive deposition of extracellular matrix (ECM) proteins^1^, particularly collagen-1 alpha. Hepatic injury, apoptosis and release of damage-related phenotypes by hepatocytes lead to activation of both hepatic stellate cells (HSCs), lymphocytes and macrophages, which in turn promote trans-differentiation of HSC and activation of myofibroblasts by production of pro-inflammatory and pro-fibrotic cytokines^2^. Upon activation, HSCs, the main source of extracellular matrix production, are transformed from their quiescent state into myofibroblast-like cells and directly start to produce collagen^3^. During this phenotypic transformation, they lose their intracellular vitamin A stores and express alpha-Smooth Muscle Actin (α-SMA, commonly known as actin alpha 2, *Acta2*), platelet-derived growth factor receptor beta (PDGFRB) and secrete collagen-1-alpha (COL1A1)^4^. Parallel to these events, the process of collagen-associated fibrogenesis is characterized by a decreased activity of matrix-degrading metalloproteinases (MMPs) and enhanced activity of tissue inhibitors of metalloproteinases (TIMPs), which are also produced by activated HSCs^5^. Respectively, collagen deposition and accumulation distort the normal hepatic architecture by forming a network of interconnecting fibrous barriers and lead to persistent and progressive impairment of the main metabolic and detoxification functions of the liver.

Curcumin has been proven in hundreds of studies to have many beneficial effects including its potency as anti-inflammatory, antioxidant, antitumor, antiproliferative, signaling pathways- and immunomodulatory, and neuro-, hepato-, and nephroprotective^6–15^. The anti-inflammatory effect of curcumin was reported to be due to its ability to regulate different inflammatory signaling pathways, with the subsequent inhibition of the inflammatory mediator’s production and action. For example, it was reported to regulate toll-like receptors (TLR) and peroxisome proliferator-activated receptor gamma (PPARγ), which results in the inhibition nuclear factor kappa B (NF-κB), mitogen-activated protein kinases (MAPK), activator Protein 1 (AP-1) and other inflammatory mediators^16–22^. However, it should be mentioned that most of these studies are in vitro studies and have not been consistently supported through clinical trials, despite this proven experimental efficacy. The major disadvantage of curcumin is its low bioavailability that limits its therapeutic potential. Its poor solubility^23^, instability^24^, poor intestinal absorption so that > 90% of the ingested curcumin is excreted in stool^25^, rapid metabolism, and rapid elimination from the bloodstream are responsible for this poor bioavailability^26,27^.

Nanotechnology may help overcoming these pharmacokinetic limitations due to curcumin insolubility and bioavailability, improving its potency to treat liver fibrosis. Of particular interest, green biosynthesized nanoparticles using reducing metabolites from microorganisms and plant-derived products comprise a better strategy to achieve cheap, less health and environmental hazardous products than artificially physical or chemical manufactured nanoparticles^28–30^. For the biosynthesis of metallic nanoparticles, bacteria are advantageous because they grow fast, able to survive at concentrations of metal ions, are safe and manipulated easily^31,32^, and their metabolites are able to reduce metallic ionic form into metal nanoparticles easily. Of these microorganisms, members of Streptomyces actinobacteria grow worldwide in soil of different environments, and are widely utilized in the industry to produce antibiotics, enzymes, and other bioactive products of commercial value. Metabolites of these microorganisms have also been recruited as reducing and capping agents to biosynthesize metal nanoparticles. There are many studies about the AgNP biosynthesis from different Streptomyces species, including *S. hygroscopicus*^33^, *S. albogriseolus*^34^, *S. albidoflavus*^35^, *S. griseorubens*^36^, and *S. catenulae*^37^. In this context, our previous study^30^ was the only one that recruited *S. malachiticus* in the green synthesis of silver nanoparticles. Furthermore, coating of those biosynthesized metal nanoparticles with non-toxic polymers like chitosan was found to reduce, but does not prevent, this toxicity, even on the fetal and placental level.

In the present study, a green curcumin nano delivery system was synthesized and recruited to treat a mouse model of liver fibrosis. This system is composed of green silver nanoparticles (AgNPs) biosynthesized by using *Streptomyces malachiticus* as characterized and published in our previous work^30^. These AgNPs were coated with chitosan, in which curcumin was embedded. These green-synthesized curcumin/chitosan-coated AgNPs were used to treat a CCl_4_-induced mouse liver fibrosis model to evaluate the role of green AgNPs in liver fibrosis and whether an improvement in the curcumin anti-inflammatory action occurs.

## Material and methods

### Biosynthesis of silver nanoparticles

*Streptomyces malachiticus* was used in the synthesis of AgNPs. The organism was grown in starch nitrate agar medium at 30 °C and grown discs were inoculated into starch-nitrate broth medium and incubated to obtain the produced metabolites. Silver nitrate (10 mM) was incubated with the obtained *S. malachiticus* metabolite, then the reaction flask was exposed to direct sunlight irradiation for 20 min. AgNPs biosynthesis is indicated by turning the color from colorless into brown due to excitation of surface plasmon resonance and reduction of silver ions by the reducing agents in *S. malachiticus* metabolites to silver metal. Details of this biosynthesis and characterization of the produced nanoparticles, including ultraviolet (UV)-visible spectral analysis, zeta potential, analysis of Fourier Transforms Infrared Spectroscopy (FTIR), and TEM analysis were published before^30^.

### Coating of AgNPs

Chitosan solution was prepared by dissolving chitosan (50,000–190,000 Da, 75–85% deacetylated, Sigma-Aldrich) in 0.5% acetic acid in a wt/v concentration of 1% with continuous stirring for 12 h. This transparent solution was then mixed with AgNPs in a proportion of 28:1 (chitosan to AgNP) for 12 h at room temperature.

Curcumin solution was prepared by dissolving curcumin in absolute ethanol (10 mg/ml) and then diluted with the acetic acid solution to reach a concentration of 5 mg/ml in 0.5% acetic acid. The chitosan-curcumin mixture was produced by dissolving chitosan in this solution to a final concentration of 1%, as forementioned. The resultant yellow mixture solution was then mixed with AgNPs in a proportion of 28:1 (chitosan-curcumin mix to AgNP) for 12 h at room temperature.

### Fibrosis model and animal grouping

Sixty male 8-week-old mice weighing ~ 25 g have been used in this study. Mice were housed with ad libitum food and water in standard conditions of temperature, humidity, and a light/dark cycle of 12:12 h. To induce liver fibrosis, 50 mice were repeatedly intraperitoneally injected with carbon tetrachloride (2 ml CCl_4_/kg b.w.) dissolved in corn oil in a ratio of 1:1 twice a week (Mondays and Fridays) for 5 weeks [3138]. The rest 10 mice received the vehicle corn oil only and served as a negative control group.

The 50 CCl_4_-treated mice were divided into 5 groups: the first 10 mice served as positive (CCl_4-_induced liver fibrosis) group; the second 10 liver fibrotic mice received non-coated AgNPs; the third 10 mice received chitosan-coated AgNPs; the fourth 10 mice received curcumin only; and the fifth 10 mice received curcumin/chitosan-coated AgNPs.

In general, several studies applied AgNP with variable doses, routes of administration and duration of administration. Doses in the literature were found to range from 250 ng/kg body weight^39^ to 120 mg/kg body weight^40^. Number of doses also ranged from 1 dose^41^ to 56 times^40^. Routes of administration varied between oral^42,43^, intraperitoneal^41,44^, subcutaneous^45^ and intravenous^40,46,47^. In the present study, mice received an intraperitoneal dose of 20 mg/kg body weight once weekly for 5 weeks. Thus, a 25 g mouse received a total ip. dose of 2.5 mg AgNPs. Mice groups were injected with different nanoparticles in the middle day (Wednesdays) between the 2 weekly CCl_4_ doses. The curcumin-treated group received an ip. dose of 50 mg/kg once weekly for 5 weeks, similar to the nanoparticle groups. Animal experiments have been approved by Damietta University board and comply with the ARRIVE guidelines and were carried out in accordance with the U.K. Animals (Scientific Procedures) Act, 1986 and associated guidelines, and EU Directive 2010/63/EU for animal experiments.

### Quantitative RT-PCR analysis of fibrogenic genes

Total RNA was extracted from livers of different groups using easy-RED Total extraction kit (Intron, South Korea). Reverse transcription was performed using first-strand HiSen-Script cDNA Synthesis Kit (Intron, South Korea). For reverse transcription, 2 μg of the total RNA was reverse transcribed to first strand complementary DNA in 25 μl reactions^48^. The resulting cDNA was amplified using a SYBR-Green PCR Master Mix (SensiFast, Bioline) and detected with a Real-time PCR system (Stratagene-Mx3000P) according to the manufacturer’s instructions. RT-PCR analysis for gene expression pattern of *Col1a1, Acta2, Pdgfrb*, and *Timp1*, in addition to β-actin as a housekeeping gene was performed using specific multiple exons-spanning primers listed in Table 1. Relative quantification was performed ΔΔCt method as published before^49^. The relative mRNA abundance of target genes was normalized to the expression of housekeeping *β-actin.* Data of qPCR are presented as fold difference from data of the negative control group, considering the control values as 1.

**Table 1:** Primers used for RT-PCR nucleotide amplifications.

Gene	Accession number	Forward primer	Reverse primer	Product size (bp)
*Col1a1*	NM_007742.4	GAGCGGAGAGTACTGGATCG	GTACTCGAACGGGAATCCATC	205
*Acta2*	NM_007392.3	TCTATGTGCTGTCCCCCTCT	ATCTCACGCTCGGCAGTAGT	208
*Pdgfrb*	NM_001146268.1	CTGTCCGTGTTATGGCTCCT	TGTCAGCACACTGGAGAAGG	204
*Timp1*	NM_001044384.1	TCCCCAGAAATCAACGAGAC	AGAAGCTGCAGGCACTGAT	229
*Actb*	NM_007393.5	AGCCATGTACGTAGCCATCC	TCTCAGCTGTGGTGGTGAAG	227

### Analysis of silver content

Parts of livers and kidneys of different mice groups were digested using a mixture (2:1 v/v) of nitric acid (1 M) and perchloric acid (1 M) for 3 h and then incubated at 120 °C for evaporation of the remaining acids. Samples were diluted with distilled water and used for silver metal concentration measurement by flame Atomic Absorption Spectrometer (PerkinElmer, PinAAcle 500, UK).

### Histopathological and biochemical analyses

Liver samples from all groups were fixed in neutral formol saline and processed for histological examination after staining with either hematoxylin and eosin^50^ for pathological changes, or Mason’s trichrome for fibrosis grading^51^. Blood samples were collected and serum was harvested and stored at − 20 °C until the determination of alanine aminotransferases (ALT), aspartate aminotransferases (AST), and albumin using commercially available kits.

### Molecular docking analysis

The calculated binding energies for curcumin as a ligand and crystal structures of some proteins involved in in liver fibrogenesis were determined using the bioinformatics tool Molecular Operating Environment (MOE, release 2015.10, Chemical Computing Group’s)^52^.

curcumin structure was constructed within MOE using the “Builder” feature. A Daylight SMILES notation for the compound was obtained from the Pubchem website (compound CID: 969516) and inserted into the SMILES field within the MOE Builder. The resulting structure was prepared for docking: protonated, partial charges adjusted and energy minimized.

Protein crystal structures were obtained from the Protein Data Bank (RCSB PDB: Homepage https://www.rcsb.org). The crystal structures of the proteins platelet-derived growth factor (PDGF) and beta-type platelet-derived growth factor receptor (PDGFRB) (PDB ID: 3MJG), Timp1 (PDB ID: 2J0T), Collagen alpha (PDB ID: 5CVB), actin alpha (PDB ID: 4Z94), and mouse TLR9 (PDB ID: 3WPF) were also prepared for docking by application of protonate 3D, correction of charge errors, inputting missing residues, and fixing residues with fractional occupancies.

A docking was completed for the energy-minimized curcumin with dummies receptors determined by the site finder of MOE on different nominated proteins, or cross docked with reference ligand sites using the program defaults. The five top poses generated from each site-specific dock were manually assessed. After exclusion of any pose with root-mean square deviation > 2 Å (RMSD that determines the distance difference of the obtained docking orientation from the corresponding pose of the reference ligand), only one pose with the highest score, and the lowest binding energy was determined for each ligand.

The inhibition constant (K_i_, the concentration required to produce half maximum inhibition) was calculated from the resulting binding energy (ΔG) using the Gibb’s free energy equation: K_i_ = exp(ΔG/RT), where R is the universal gas constant (1.985 × 10^−3^ kcal mol^−1^ K^−1^) and T is the absolute temperature (298.15 K)^53^.

### Statistical analysis

Data are expressed as mean ± standard error of mean (SEM). The independent one-way ANOVA was used for data analysis between diverse groups followed by student's t-test as a posthoc test, whenever ANOVA was significant. Paired comparisons were done by t-test, when the application of ANOVA was not required. The statistical significance of all data was set at *p* < 0.05.

## Results

### The nano delivery system

**Figure 1:**
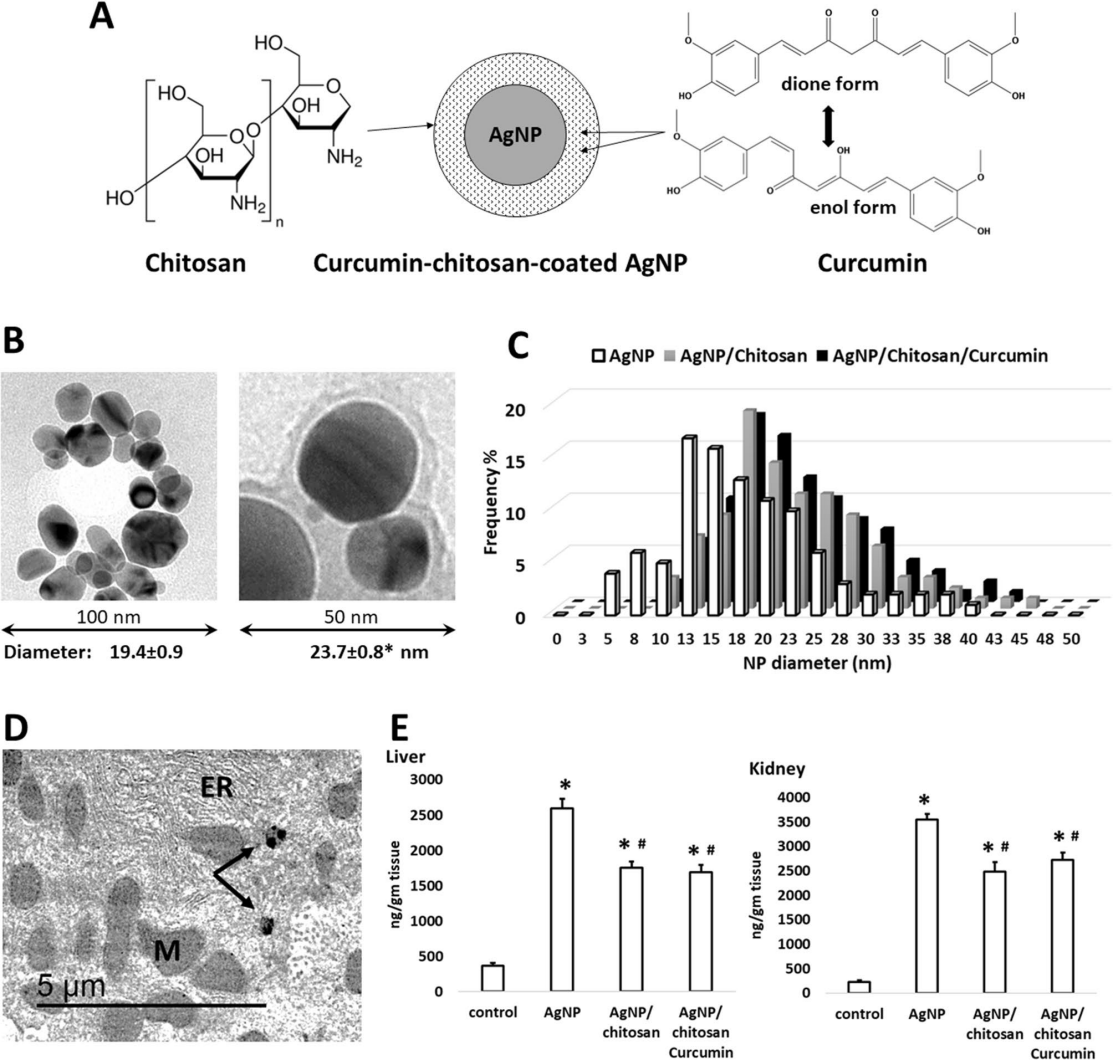
(**A**) structure and components of curcumin/chitosan-coated silver nanoparticles. This nano-delivery system consists of a core of green-synthesized AgNP surrounded by a coat of a mixture of chitosan and curcumin. The left panel shows the chemical structure of chitosan, and the right panel shows the chemical structure of both curcumin forms. (**B**) transmission electron microscopy (TEM) of non-coated (left) and coated (right) AgNPs. The diameter is expressed as mean ± SEM of n = 100 measured NP diameters. The * denotes significant difference in diameter between non-coated and coated AgNPs (t-test). (**C**) the diameter of most non-coated AgNPs ranged between 12.5 and 22.5 nm, while that of coated NPs peaked at 17.5–20 nm. (**D**) intrahepatocyte detection of AgNPs (arrows) by TEM of a liver from AgNP-treated mouse. ER, endoplasmic reticulum; M, mitochondria. (**E**) estimation of Ag content in livers and kidneys of mice treated with coated or non-coated AgNPs. Statistical analysis: ANOVA *P* < 0.05; * = significantly higher than the control values; # = significantly lower value of silver content (posthoc t-test after ANOVA).

It was sought to embed curcumin in a nano structure and use it as anti-inflammatory agent in a mouse liver fibrosis model. This green nano delivery system (Fig. 1A) is composed of a nano-core of green synthesized AgNP, which is surrounded by a degradable chitosan coat containing curcumin as a drug.

The green nano core was synthesized by metabolites of *Streptomyces malachiticus* and characterized as published in our previous work^30^. Analysis of Zeta potential of these biosynthesized AgNPs showed a turn from negative to positive peaks after chitosan coating. Ultrastructure analysis (Fig. 1B) showed nearly round AgNPs with diameters ranging from 6.1 to 44.6 nm and averaged 19.4 ± 0.9 nm (mean ± SEM). Diameter distribution is shown in Fig. 1C. Diameters of coated AgNPs ranged from 10.32 to 48.98 nm with an average diameter of 23.7 ± 0.8 nm. The diameter of coated NP included the coating thickness, and therefore, they appeared thicker than naked AgNPs. The synthesis of NP was a 2-step procedure. The first was the green synthesis of the metal core. Each preparation was then divided into 3 portions: one was left naked, one was coated with chitosan, and the third portion was coated with chitosan solution that contains curcumin. Thus, coating process added extra thickness to the original metal NP core.

The uptake of both coated and non-coated AgNPs into mouse tissues has been proven. They were observed in vesicles in hepatocytes by ultrastructural examination (Fig. 1D). Estimation of silver content in hepatic and renal tissues by atomic absorption spectrometer (Fig. 1E) revealed a significant 7 and 5 times increase in silver content in livers treated with non-coated and coated AgNPs than in the control group, respectively; and 15 and 11 times in kidneys. However, the increase was significantly lower in mice treated with coated AgNPs than that treated with non-coated NPs.

### Liver function

**Figure 2:**
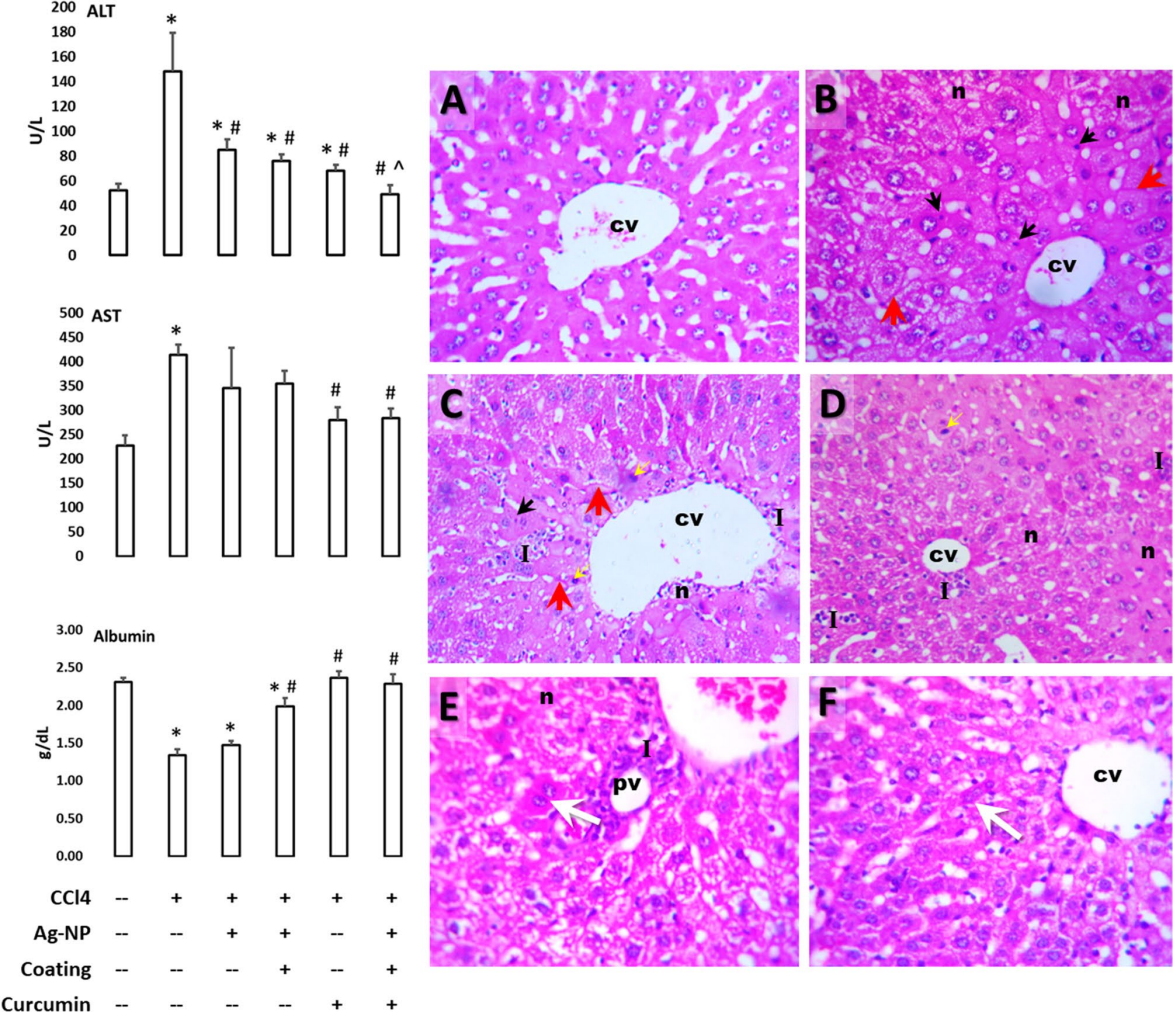
The green synthesized curcumin/chitosan-coated silver nanoparticles maintain the liver structure and function of CCl_4_-mouse fibrosis model. Mice were treated twice a week with CCl_4_ and once a week with different nano treatments or curcumin for 5 weeks. Left panel: serum levels of the hepatic enzymes AST and ALT, and albumin after different treatments as a liver function test. Statistical analysis: ANOVA *P* < 0.05; * = significantly different from the control value; # = significantly different from value of CCl4-fibrosis group; ^ = significantly lower than the value of curcumin-treated group (posthoc t-test after ANOVA). Right panel: hepatic histological changes (H&E, 200x) from negative control group (**A**) due to fibrosis induction by CCl_4_ (**B**) and treatment of this fibrosis group with AgNPs (**C**), chitosan-coated AgNPs (**D**), curcumin (**E**), or curcumin/chitosan-coated AgNPs (**F**). Abbreviations: cv, central vein; pv, portal vein; n, necrosis, I, necrotic foci with leukocytic infiltration; dark arrows show apoptotic microneuclei; red arrows, fibrotic thickenings; white arrows, binucleated hepatocytes.

Mouse liver fibrosis model was initiated by 2 × weekly CCl_4_ ip injections for 5 weeks. Concurrently, the same mice were grouped and treated once weekly with either nothing (positive control fibrosis group), AgNPs, chitosan-coated AgNPs, curcumin, or curcumin/chitosan-coated AgNPs. At the end of the fifth treatment week, sera were processed for liver function tests including AST, ALT, and albumin (Fig. 2). Treatment with CCl_4_ was characterized by significant elevations of the both liver enzymes and lower albumin level as compared to untreated controls. Only curcumin/chitosan-coated AgNPs administration led to significant protection against CCl_4_-induced changes, since it was associated with significantly lower serum liver enzyme levels and higher serum albumin level than the CCl_4_ group. Data of this treatment was similar to that of the non-treated negative control group, while variable results were obtained for other nano- and curcumin-treated groups (Fig. 2).

### Liver histopathology

At the end of the fifth treatment week, livers from different treatment groups were processed for histological examination. Microscopic observation of hematoxylin-and-eosin-stained hepatic tissue from control mice revealed normal hepatic architecture. The connective tissue in the liver indistinctly divided the hepatic parenchyma into the classic functional hepatic lobules. Each hepatic lobule demonstrated poorly defined hepatocytes cords or plates delineation especially in the midlobular and centrilobular regions. These plates comprised hepatocytes extending from the portal region to terminal hepatic venule (central vein). No sign of hepatic necrosis, steatosis, inflammation or fibrosis were observed (Fig. 2A). Sinusoidal capillaries (sinusoids), the vascular channels found between the plates of hepatocytes appeared to radiate out from a small central vein. In between the sinusoidal lining cells and hepatocytes lies the space of Disse, containing Küpffer cells, hepatic stellate cell, and pit cells (natural killer lymphocytes) were presented. Portal regions comprising four components: arterioles, venules, bile ductules, and lymphatics were less apparently detected.

Hepatic sections from CCl_4_-treated mice (Fig. 2B) revealed marked massive hepatic architectural lesions. These lesions were characterized by lytic necrosis within midlobular and centrilobular regions; necrotic hepatocytes containing acidophilic hyaline inclusions; ballooning degeneration, thickened wall of central vein, dilation and congestion of portal vein, massive periportal inflammatory cellular infiltration and fibrosis., intracellular micro- and macrovacuoles, clusters of hypertrophic Kupffer cells and macrophages, hypertrophic hepatic stellate cells, marked variation in the size, shape, and staining qualities (pleomorphism) of hepatocytes.

Hepatic tissue injury was similar to or even more massive than the previous group in CCl_4_ and non-coated AgNP-treated mice. The hepatic architectural lesions were manifested by pale staining foci of hepatic necrosis within midlobular and centrilobular areas, while periportal necrotic hepatocytes containing acidophilic hyaline inclusions. Bridging bands of hepatocytes with ballooning degeneration connected the portal tract to the central hepatic venule or the adjacent hepatic portal area. Thickening wall of central vein, dilation of portal vein, massive extended periportal inflammatory cellular infiltration, intracellular micro- and macrovesicles, hypertrophic Kupffer cells and hepatic stellate cells in dilated sinusoids, marked variation in the size, shape, and staining qualities (pleomorphism) of hepatocytes were also observed (Fig. 2C).

Hematoxylin and eosin-stained hepatic tissue from CCl_4_ and chitosan-coated AgNP-treated mice (Fig. 2D) exhibited less hepatic architectural lesions when compared with the group treated with non-coated AgNPs. The lesions have been manifested by pleomorphism of the hepatocytes resulted from combination of diffuse hepatocellular degeneration and hepatocellular regeneration, pale-stained necrotic hepatocytes within some centrilobular area, necrotic hepatocytes containing acidophilic hyaline inclusions; some hepatocytes showed ballooning degeneration or apoptosis, dilation of portal vein, intracellular microvesicles, hypertrophic Kupffer cells and hepatic stellate cells in dilated sinusoids. Some signs of hepatic regeneration were observed, including binucleation of hepatocytes and clusters of small basophilic regenerating hepatocytes.

Similar results were observed in H&E-stained hepatic tissue from CCl_4_ and curcumin-treated mice (Fig. 2E). Changes were manifested by hepatocytes necrosis and ballooning degeneration within centrilobular, midlobular and periportal areas, some necrotic hepatocytes containing acidophilic hyaline inclusions; congestion and dilation of portal vein, periportal inflammatory cellular infiltration, some intracellular micro- and macrovesicles, hypertrophic Kupffer cells and hepatic stellate cells in dilated sinusoids. Signs of hepatocellular regeneration were also detected.

Inclusion of curcumin in the chitosan coat of the AgNPs led to minor hepatic architectural lesions in CCl_4_-mice and exhibited maintenance of normal hepatic architecture (Fig. 2F). Lesions were manifested by some pleomorphism of the hepatocytes resulting from a combination of diffuse hepatocellular ballooning degeneration and hepatocellular regeneration. Some hepatocytes showed apoptosis, dilation of central hepatic venule and portal vein, and microvesicles. The detected sign of hepatic regeneration was characterized by binucleation and multinucleation of hepatocytes and small clusters of basophilic regenerating hepatocytes.

### Distribution of active fibrosis

**Figure 3:**
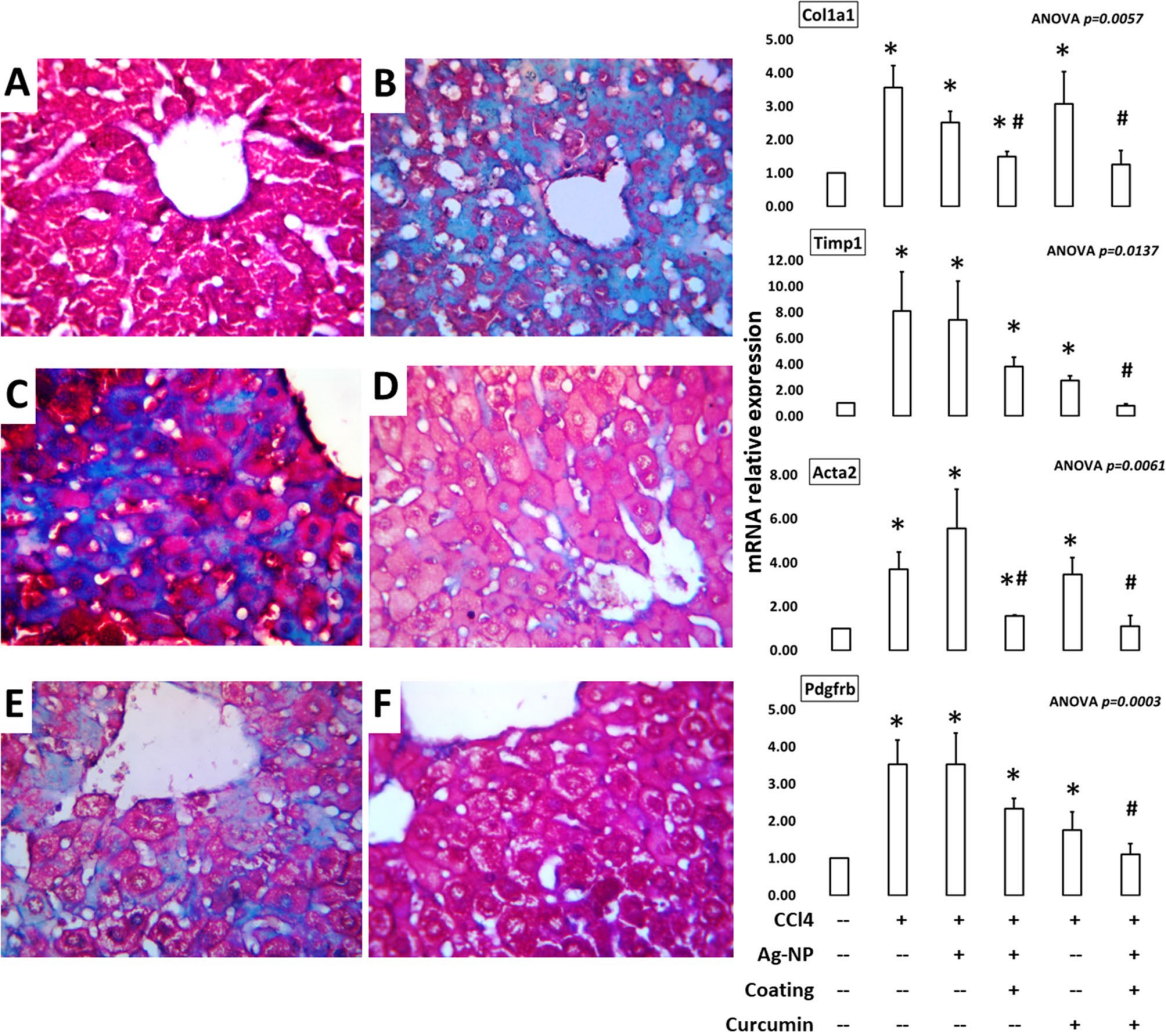
Treatment with the green synthesized curcumin/chitosan-coated silver nanoparticles prevents the development of fibrosis in CCl_4_-mice liver. Mice were concurrently treated twice a week with CCl_4_ and once a week with different nano treatments or curcumin for 5 weeks. Left panel: Mason’s trichrome showing hepatic fibrosis (blue-stained) after induction by CCl_4_ (**B**–**F**) and treatment with AgNPs (**C**), chitosan-coated AgNPs (**D**), curcumin (**E**), or curcumin/chitosan-coated AgNPs (**F**). (**A**) represents the negative control group. Right panel: Quantitative PCR analysis for genes involved in fibrogenesis of hepatic cells. RNA was extracted from mice livers after different treatments and transcribed to cDNA. RT-PCR was applied using these cDNAs and the primer pairs listed in Table 1. The shown data are averages of three separate experiments. Statistical analysis: ANOVA *P* < 0.05 for all genes; * denotes significantly different value from the control one; # denotes significantly different from value of CCl4-fibrosis group (posthoc t-test after ANOVA).

After 5 weeks of CCl_4_-, curcumin-, and nano-treatments, liver sections were subjected to Masson’s trichrome staining to highlight the occurrence and distribution of reactive fibrosis as a result of CCl_4_-induced liver injury. In control liver sections, no collagenous connective tissue between lobules, in the portal space and around the central vein was observed (Fig. 3A). In CCl_4_-treated group (Fig. 3B), signs of massive hepatic fibrosis were detected. Hepatic section revealed extensive collagenous deposits predominantly in the centrilobular zone, pericellular and perisinusoidal space throughout the hepatic lobules. In CCl_4_ and non-coated AgNP-treated mice (Fig. 3C), similar results were obtained. Hepatic sections revealed collagenous deposits predominantly in the centrilobular and periportal zones, pericellular and perisinusoidal space throughout the hepatic lobules.

An enhancement has occurred on treatment of CCl_4_ mice with chitosan-coated AgNPs (Fig. 3D), In Masson’s trichrome stained liver sections, little signs of hepatic fibrosis were detected. Hepatic section revealed few collagenous deposits predominantly in the pericellular and perisinusoidal space throughout the hepatic lobules. The pericellular fibrosis was seen prominently around ballooned hepatocytes. In CCl_4_ and curcumin-treated group (Fig. 3E), moderate signs of hepatic fibrosis were detected. Hepatic section revealed moderate amounts of collagenous connective tissue throughout hepatic lobules. In each lobule, a delicate stroma of reticular fibers forms a network within the perisinusoidal space.

Finally, in the curcumin/chitosan-coated AgNP-treated group (Fig. 3F), Masson’s trichrome staining showed no signs of hepatic fibrosis, despite concurrent treatment with CCl_4_. Hepatic section revealed normal amount of collagenous connective tissue throughout the hepatic tissue.

### Expression of genes involved in liver fibrosis

Next, we sought to determine which fibrogenic genes are affected by the treatment with nanoparticles and curcumin. For this purpose, RNA was extracted from livers of different groups and processed for qPCR using primers for collagen type 1 alpha 1 (*Col1a1*), alpha-smooth muscle actin (alpha-SMA, aka *Acta2*), platelet-derived growth factor receptor beta (*Pdgfrb*), tissue inhibitor of metalloproteinases 1 (*Timp1*), and the housekeeping beta-actin (*Actb*) as listed in Table 1. The quantitative RT-PCR results (Fig. 3, right panel) demonstrated an upregulation of all tested genes upon fibrosis induction by CCl_4._ Concurrent treatment of this model with AgNPs did not reduce this gene upregulation. The result of *Acta2* gene expression demonstrates that AgNPs tend to act in the direction of fibrogenesis. Also, treatment with curcumin neither suppressed the expression level of collagen nor alpha actin genes. Positively, encapsulation of AgNPs was able to downregulate the expression of both genes, as compared with their expression in CCl_4_ group. However, their expression levels were still significantly higher than that in the non-treated group. The results demonstrate also that embedding of curcumin in the chitosan coat of AgNPs was able to maintain the expression of all of the above-mentioned genes at the control level. In all treatments, the expression of fibrogenic genes was associated with the degree of development of liver fibrosis detected by Mason’s trichrome staining.

### Molecular docking

**Figure 4:**
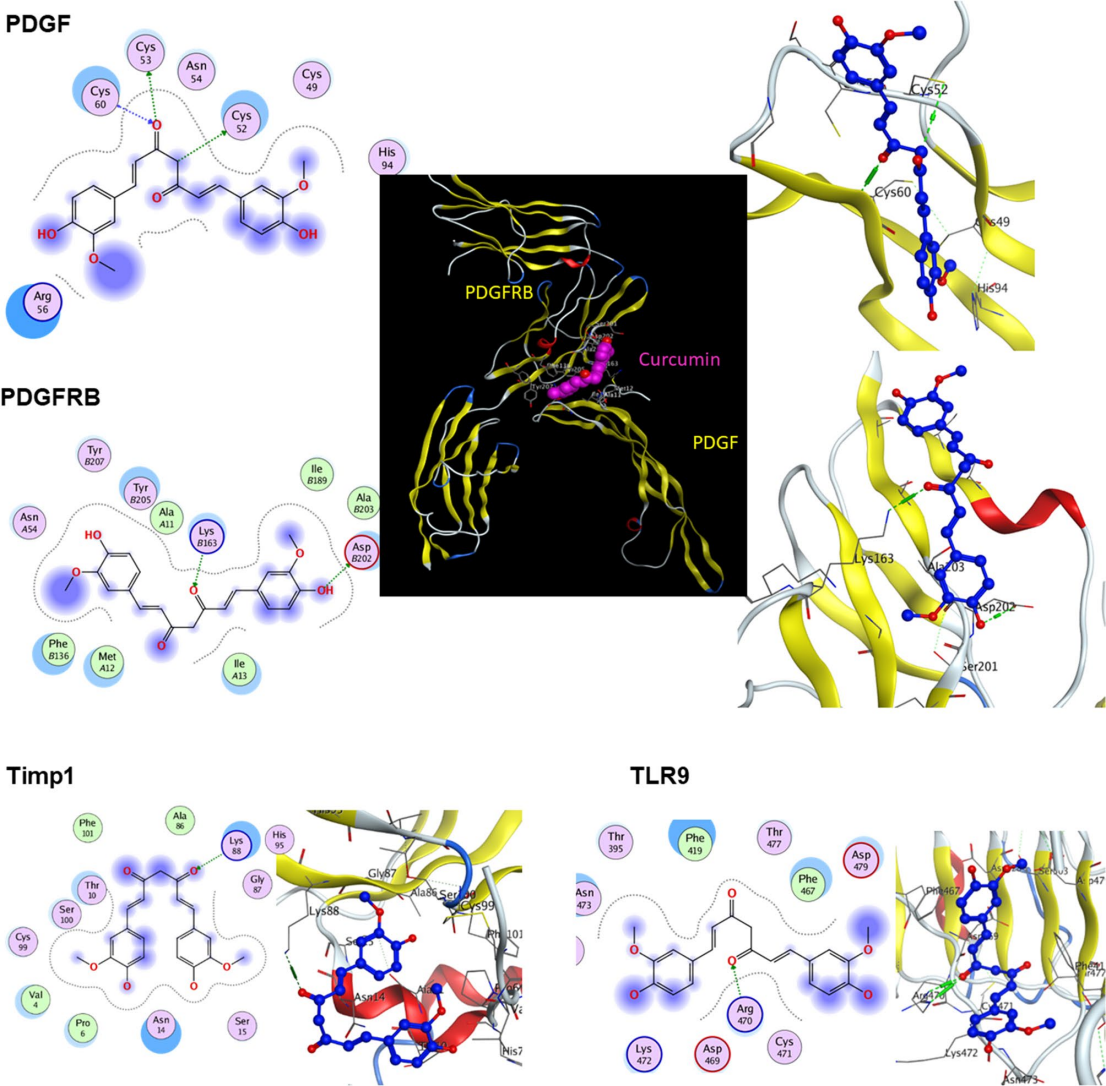
Molecular docking of curcumin in complex with different proteins involved in liver fibrogenesis. Left images are the structure and the 2D interaction between the curcumin and the receiving amino acid residues in the protein. Right images are the 3D docking position for curcumin (blue) binding with different proteins. Hydrogen bonds are presented in green. The middle black-backgrounded image is the 3D docking position for curcumin (magenta) binding with and positioned in the binding site between both PDGF and its receptor PDGFRB.

It is concluded from the RT-PCR data above that curcumin tend to downregulate specific genes as *Timp1* and did not affect the expression of other as *Col1a1* and *Acta2*. Therefore, it was attempted to address interactions of different proteins contributed to liver fibrogenesis and curcumin using in silico approach. Molecular docking results revealed that curcumin could not dock into collagen alpha or actin alpha, while it binds to Timp1, PDGF, PDGFRB, and TLR9 proteins with variable docking scores and binding affinities. However, it is not docking score that decides the strong interaction between the protein and ligand. Strong binders are determined according to the individual binding energy, taking in consideration that all of them has acceptable binding scores. The interactions between curcumin and these fibrogenic proteins are presented in Table 2 and Fig. 4. The most favorable docking pose that has the lowest binding energy was presented here and selected for the intramolecular interaction analysis.

**Table 2:** Molecular interactions between curcumin and some proteins involved in liver fibrogenesis.

Protein	Score	RMSD (Å)^a^	Receptor residue^b^	Curcumin interaction^c^	Binding Distance^d^	binding energy (kcal/mol)	K_i_ (µM)^e^
Timp1	− 5.55	1.11	Lys88	H-acceptor	3.09	− 4.6	421.15
PDGF	− 5.82	1.43	Cys60	H-acceptor	3.00	− 4.4	590.47
PDGFRB	− 5.34	1.75	Lys163	H-acceptor	2.98	− 6.1	33.40
TLR9	− 6.30	1.99	Arg470	H-acceptor	2.86	− 6.0	39.54

^a^RMSD is the distance difference (Å) of the obtained docking orientation from the corresponding pose of the reference ligand;

^b^In some cases, more than 1 residue participates in the binding process (Fig. 4). The residue mentioned here is the most prominent with the shortest binding distance and lowest binding energy;

^c^In some cases, some other ionic and van der Waal bonds may exist. The one mentioned here is that corresponding to the lowest binding energy and shortest binding distance;

^d^The binding distance (Å) between the reactive protein residue and the ligand binding atom;

^e^The inhibition constant (K_i_) is the concentration required to produce half maximum inhibition and was calculated from the resulting binding energy (ΔG) using the Gibb’s free energy equation: K_i_ = exp(ΔG/RT), where R is the universal gas constant (1.985 × 10^−3^ kcal mol^−1^ K^−1^) and T is the absolute temperature (298.15 K).

The docking analysis reflected binding energy of − 6.1 kcal/mol for PDGFRB-curcumin interaction. In this complex, curcumin was a hydrogen bond acceptor from Lys163 and donor to Asp202. The calculated inhibition constant (K_i_) was 33.4 µM. Curcumin could also dock to PDGF with binding energy of − 4.4 kcal/mol. This interaction consisted of 3 hydrogen bonds between curcumin and Cys52, Cys53 (donor) and Cys60 (accepting) of the protein PDGF. However, the lower interaction energy observed for the receptor PDGFRB rationalizes a tighter binding of curcumin than that with the ligand protein PDGF. When docked with the PDGF-PDGFRB complex model, curcumin was found to dock itself between both proteins. To form this PDGF-PDGFRB complex, it was reported that Glu15 in PDGF forms a salt bridge with Lys163 of PDGFRB^54^. Thus, curcumin binding to Lys163 of PDGFRB may inhibit the formation of this PDGF-PDGFRB complex, which signaling enhancement represents a hallmark in the development of liver fibrosis.

Good docking results were obtained for the interaction between curcumin and Toll-like receptor 9 (TLR9), with binding energy of − 6.0 kcal/mol and calculated inhibition constant (K_i_) of 39.54 µM. TLR9 plays a crucial role in liver fibrogenesis, since the activation of hepatic stellate cells is partially mediated by the interaction of hepatocyte DNA with TLR9 expressed in HSCs and the subsequent proinflammatory response^55,56^. It was reported that TLR9 knockout mice demonstrated less, or even protected from, liver fibrosis than controls^57,58^. Curcumin binds to Arg470 residue in TLR9 with a strong hydrogen bond.

Curcumin was found also to dock into Timp1 protein with lower affinity than that with PDGFRB or TLR9. The binding energy for this interaction was − 4.6 kcal/mol and calculated inhibition constant (K_i_) of 421.15 µM, and the receptor residue for curcumin in Timp1 was Lys88.

## Discussion

Curcumin is a natural compound with a variety of biological activities and great potential for treatment of many diseases, including liver diseases^9^. Yet, its practical application is still limited due to its inherent problems. Therefore, it would be valuable to provide new ideas to develop beneficial curcumin preparations to overcome these problems. In this study, a nano system composed of a nanocore of eco-friendly synthesized AgNPs surrounded by a coat of curcumin-chitosan mixture has been designed to overcome the curcumin problem of insolubility and to make it more bioavailable for the treatment of mouse liver fibrosis model. Nanocurcumin was previously reported to disperse freely in aqueous media in the absence of any surfactants^59^, increasing curcumin bioavailability and overcoming its physiological barriers^60^. In aqueous conditions, curcumin nanoparticles were found to be several folds more effective on cancer cells than native curcumin^61^. The designed green synthesized nano-system in the present study is more advantageous than chemically or physically manufactured NPs. This AgNP-based nano system, made by the metabolite of *S. malachiticus*, has lower production cost, less environmental hazards using cheap local resources, low reaction time and reaction temperature (at room temperature in the presence of sunlight within minutes), higher biosafety, no specific equipment, no energy consumption, reducing and capping agent, and less physiological toxicity^30,62^. chitosan (50,000–190,000 Da, 75–85% deacetylated) was used in the present study for the coating of AgNPs. In fact, chitosan with different MW, deacetylation and also salt level may affect the physicochemical characters, stability, drug releasability and also silver releasability and subsequent in vivo toxicity of the applied nanoparticles. These parameters are important to study, and optimization of the system should be done, but this is out of scope of this manuscript, which focused only on the application of a nano-delivery system on a model of liver fibrosis. We applied this MW chitosan based on the previous publications related to animal trials and in vivo experiments in the literature^63–65^. This low molecular weight chitosan may be preferred in drug delivery for several reasons. For example, higher molecular weight chitosan produces larger size nanoparticles^66^, which may affect permeability and drug delivery into cells. Nanoparticles with low molecular weight chitosan was reported to show higher transfection and delivery efficiency^67,68^. Also, the amount of the released silver was found to be less in application of lower than higher MW chitosan (800 kd), which was characterized by a huge amount of the released silver^69^. Release of ionic silver causes more in vivo toxicity. Thus, for application as antibacterial nanoparticles or for in vitro cytotoxicity purposes, higher molecular weight may be preferred, since higher release of silver may increase cytotoxicity.

The present engineered nanoparticles, containing curcumin, are thought for efficient penetration into hepatocytes. In our previous work^30^, it has been proven that these nanoparticles penetrate even into the fetus through the placenta. In the present study, the hepatocellular uptake of silver nanoparticles has been proven by the observation by TEM, and, as seen in Fig. 1, silver content increased > 7 and > 5 times the control level in livers treated with non-coated and coated AgNPs, respectively. The results related to AgNPs penetration into hepatocytes are in agreement with other studies in vivo and in vitro^70–72^. This cellular internalization has been also reported for other metal/chitosan/curcumin nano systems. Curcumin loaded into chitosan/gold nanoparticles showed higher cellular uptake in huh7 and MCF7 cell lines compared to native curcumin^73^.

The intracellular uptake takes place by the processes of phagocytosis, micropinocytosis and clathrin-mediated endocytosis^74^, depending on their size and surface charge. in the cytoplasm, curcumin molecules will be released from the biodegradable chitosan coat and interacts with the intracellular salty conditions. Alternatively, nanoparticles remain trapped in lysosomes and endosomes^75^, where they are exposed to high ionic conditions during digestion^72^. These lysosomal digestive conditions release curcumin from chitosan coat into the cytosol. Thus, the hydrophobicity, which represents the main challenge limiting the medical application of curcumin, was overcome by its embedding in this chitosan coat in a nanoparticle scale that is able to penetrate the cell lipid membrane and be intracellularly delivered. On entering into the hepatocytes, the core AgNP will accompany the surrounding curcumin coat, enhancing its membrane permeability, increasing intrahepatocyte curcumin bioavailability and allowing for its anti-inflammatory action. Other employed solvents/chemical mediators to solvate and treat with native curcumin such as dichloromethane and dimethyl sulfoxide showed limited pharmacologic action and may cause dangerous cytotoxic effects^76,77^.

Growing data suggests that most ingested curcumin is excreted^25^ and the rest is rapidly eliminated from the bloodstream^26,27^, so that there is no curcumin left to reach the target tissues to render a therapeutic effect. Meanwhile, many studies, including ours, have reported that the treatment with AgNPs leads to their accumulation in many organs, preferentially liver and kidneys^78,79^, which are the first organs to actively metabolise from the blood and excrete such compounds. The present study demonstrates a significant increase in content of silver of livers treated with naked and coated AgNPs. This increase in silver content was reported to be toxic to the liver^30,78–80^. In the present study, treatment of CCl_4_-induced fibrotic liver with naked AgNPs tended to increase the damaging effect of CCl_4_ as shown by the significant histopathological and biochemical changes of the liver enzymes AST and ALT, and albumin. On the positive side, encapsulation of these AgNPs with chitosan was reported to reduce this toxicity^30^, as shown also in the present study. It was expected that adding curcumin to this chitosan coat will further minimize this toxicity.

Pathogenesis of liver fibrosis is associated with many etiologies including alcoholic liver disease, non-alcoholic fatty liver diseases and non-alcoholic steatohepatitis, and chronic viral hepatitis. Liver fibrosis can lead to liver cirrhosis, and ultimately to organ failure and death, if left untreated^3^. In response to chronic hepatic injury, fibril-forming, especially collagen type 1, and other matrix proteins accumulate, and fibrosis is driven by this accumulation of extracellular matrix (ECM) components accompanied by a failure of the mechanisms responsible for matrix turnover. Accumulation of ECM activates hepatic stellate cells HSC and further amplify fibrosis by activating positive feedback pathways^81^. Growing data suggests that specific intrahepatic proteins are constitutively activated during liver fibrogenesis. In healthy state, accumulation of ECM is controlled by matrix metalloproteinases (MMPs). During fibrogenesis, MMPs are inhibited by the tissue inhibitors of matrix metalloproteinases TIMP-1 and TIMP-2. Downregulation of TIMPs in HSCs was reported to reverse fibrosis^82^. In the present study, *Timp1* was upregulated by CCl_4_ and downregulated by curcumin. Accumulation of ECM causes also production of cytokines including, among others, platelet derived growth factor (PDGF), transforming growth factor β (TGF-β), hepatocyte growth factor (HGF), connective tissue growth factor (CTGF), and tumor necrosis factor-α (TNF-α)^83^. Liver injury provokes sinusoidal endothelial cells also to produce fibronectin, TGF-β1 and PDGF and contribute to HSC activation^84^. Hepatocyte apoptosis in liver injury releases DNA, which interacts with TLR9 produced by HSCs^56^. Activation of HSCs leads them to produce alpha smooth muscle actin (α-SMA) platelet-derived growth factor β-receptor (Pdgfrb)^4^ and further secrete collagen-1 alpha. The present study demonstrated the upregulation of the expression of these 3 genes by CCl_4_ treatment, accompanied by the increase of fibrosis as proved histologically. These gene upregulations and the fibrotic effect were prevented by the concurrent treatment with nano-curcumin. In fact, some of these genes such as *Col1a1*, was reported to be a biomarker and putative therapeutic target for liver cell carcinogenesis and metastasis^85^. Thus, downregulation of these genes suggests the therapeutic utility of curcumin in fibrotic liver diseases.

In response to many of the fibrogenic mediators mentioned above, HSCs produce reactive oxygen species (ROS) that can upregulate the expression of fibrosgenic genes such as *Col1a1* and *Timp1* in HSCs^86^. Curcumin was reported to control multiple enzymes involved in the ROS metabolic pathway as curcumin-binding targets^87–89^. This control may explain the downregulation of these genes by nano curcumin in this study.

In the present study, the anti-inflammatory and anti-fibrosis effect of curcumin was suggested to be due to a direct binding of curcumin with some of the fibrosis-associated proteins mentioned above. To investigate the anti-inflammatory property of curcumin in silico, it was previously reported that curcumin and analogues could dock successfully into the TGF-β mentioned above^90^ and its receptor ALK5^91^, TNF-α^92^ cox2^90,93^, and phosphodiesterase 4 (PDE4)^94^. In the present study, a direct physical binding between curcumin and the fibrogenic proteins PDGF and its receptor PDGFRB, Timp-1 and TLR-9 was demonstrated with variable affinities. We found also, in silico, that curcumin can disrupt the physical association of PDGF and its receptor, which may lead to inhibition of liver fibrogenesis. Taking together, these novel molecular docking results suggest that the curcumin anti-liver fibrotic action can be attributed to the inhibition of these fibrosis-associated proteins.

In conclusion, a nano delivery system composed of a nanocore of eco-friendly silver nanoparticle biosynthesized by the metabolite of the actinobacteria *Streptomyces malachiticus,* and coated with curcumin-chitosan mixture has been designed in this study to overcome the curcumin problems of insolubility and permeability, and to make it more bioavailable for the treatment of a mouse liver fibrosis model. This nano delivery system is cost-effective, easy to be prepared and delivered to the liver. It helped enhancing curcumin membrane permeability, increasing its intrahepatocyte bioavailability and allowing for its anti-fibrosis action. This permeability into hepatocytes has been proven by a direct observation of AgNPs in the hepatocytes by TEM and silver level estimation in the liver tissue. These green-synthesized chitosan-coated silver NPs were used to treat a CCl_4_-induced mouse liver fibrosis model to evaluate the role of green AgNPs in liver fibrosis and whether an improvement in the curcumin anti-inflammatory action occurs. After delivery into hepatocytes and release from the biodegradable chitosan coat, curcumin in this form was found to be efficient as anti-hepatic fibrosis drug, maintaining the hepatic architecture and function in the studied fibrosis model. This curcumin anti-hepatic fibrosis efficacy can be attributed to its inhibitory role through a direct binding to fibrosis-mediating proteins such as PDGFRB, Timp-1, TLR-9 and TGF-β.

## Data Availability

All data generated or analyzed during this study are included in this published article.
